# Finite Element Investigation of Patellofemoral Contact Mechanics: Influence of Tibial Tuberosity Lateralisation and Trochlear Dysplasia on Extensor Mechanism Stability

**DOI:** 10.3390/life15091442

**Published:** 2025-09-15

**Authors:** Georgian Iacobescu, Antonio-Daniel Corlatescu, Loredana Iacobescu, Bogdan Serban, Catalin Cirstoiu

**Affiliations:** 1 Department of Orthopaedics and Traumatology, Carol Davila University of Medicine and Pharmacy, 050474 Bucharest, Romania; georgian.iacobescu@umfcd.ro (G.I.); antonio.corlatescu@gmail.com (A.-D.C.); bogdan.serban@umfcd.ro (B.S.); catalin.cirstoiu@umfcd.ro (C.C.); 2 Department of Orthopedics, University Emergency Hospital, 050098 Bucharest, Romania; 3 Department of Cardiology, University Emergency Hospital, 050098 Bucharest, Romania

**Keywords:** patellofemoral instability, trochlear dysplasia, tibial-tuberosity transfer, finite-element analysis, contact pressure, medial patellofemoral ligament

## Abstract

Background: Patellofemoral instability arises from the interplay between trochlear morphology and malalignment of the extensor vector. Although each factor is individually well described, their combined mechanical effects have not been quantified within a single finite element framework. Objective: To investigate how lateral trochlear inclination (LTI) and tibial tuberosity position interact to influence patellofemoral contact mechanics and stability across clinically relevant knee flexion angles. Methods: A subject-specific finite element model of the femur–patella–tibia complex was reconstructed from high-resolution CT data. Cortical and cancellous bone, patellar cartilage, the MPFL, and patellar tendon were included. Three trochlear morphologies were simulated (LTI = 15°, 10°, 5°) under native alignment (Case A) and after 10 mm lateral tibial tuberosity translation (Case B). Flexion at 30°, 60°, and 90° was imposed via solver-applied tibial displacement. Primary outcomes were contact pressure, contact area, MPFL stress, and lateral patellar translation. Instability was defined as >5 mm lateral translation or >50% reduction in contact area, consistent with the biomechanical literature. Model convergence (<5% variation) and validation against cadaveric pressure data were performed; a sensitivity analysis tested material property variation (±15%). Results: The native model reproduced peak pressures (3.6 MPa at 60°) within 9% of experimental benchmarks. Decreasing LTI enlarged the contact patch and lowered mean pressures (−18%) but increased MPFL stress (+37%). Tibial tuberosity lateralisation reduced mean pressures further (−25%), yet, when combined with shallow trochlear slopes (≤8°), produced >5 mm lateral patellar translation and near-complete loss of cartilage contact by 60°, simulating lateral dislocation. Sensitivity testing confirmed robustness to material property uncertainty. Conclusions: Shallow trochlear inclination dissipates articular load but destabilises the patella, an effect magnified by tibial tuberosity lateralisation. While these findings highlight thresholds at which stability may be compromised, they derive from a single-subject model and should be interpreted as hypothesis-generating rather than prescriptive. Broader validation across multiple geometries and loading conditions is required before clinical translation.

## 1. Introduction

Patellofemoral instability is a frequent problem in adolescent and young adult populations, with reported incidences ranging from 6 to 29 per 100,000 and a clear female predominance. Its natural history is worrisome because recurrent lateral dislocation accelerates cartilage damage and precipitates early patellofemoral osteoarthritis [1].

Current pathomechanical thinking recognises a constellation of bony and soft-tissue risk factors. The most influential osseous variables are trochlear dysplasia, patella alta, and an increased tibial tuberosity–trochlear groove (TT–TG) distance, while secondary contributors include femoral or tibial torsional malalignment and coronal plane valgus. Clinically, patients often harbour several anomalies simultaneously; one series found that 86% of unstable knees had at least two major risk factors [2,3].

Surgical strategy therefore needs to be individualised. Medialising or anteromedialising tibial tubercle osteotomy realigns the extensor vector pull, trochleoplasty deepens the groove to restore osseous containment, and medial patellofemoral ligament (MPFL) reconstruction reinstates soft-tissue restraint. Each procedure, however, alters patellofemoral contact mechanics—sometimes beneficially, sometimes detrimentally. Deepening trochleoplasty, for example, has been shown to raise retropatellar pressures in silico, whereas isolated MPFL overtension or aggressive lateral retinacular release can conversely unload cartilage at the cost of stability [4,5].

Finite element (FE) modelling has become an indispensable tool for dissecting these interactions, allowing non-invasive exploration of stress distributions that are impossible to capture in vivo with current imaging. Early FE work quantified normal pressure patterns during weight-bearing flexion, and more recent parametric studies have examined individual manoeuvres such as trochleoplasty depth, MPFL tension, and degree of lateral retinacular release. Yet no numerical study to date has evaluated, within a single framework, how trochlear dysplasia severity and tibial tuberosity lateralisation jointly influence both contact stress and the risk of mechanical disengagement across the functional flexion arc [6,7].

The purpose of the present investigation is to fill that gap. Using a subject-specific three-dimensional FE model of the extensor mechanism, we tested the hypothesis that tibial tuberosity lateralisation decreases mean patellofemoral pressure but simultaneously compromises patellar containment in proportion to the lateral inclination deficit of the dysplastic trochlea. By simulating three clinically relevant flexion angles (30°, 60°, 90°) and three grades of trochlear morphology under native and lateralised conditions, we aimed to produce quantitative thresholds that can refine surgical indications for combined bony and soft-tissue realignment procedures.

## 2. Materials and Methods

### 2.1. Overall Study Design

We developed a deterministic, quasi-static finite element (FE) analysis to observe patellofemoral contact mechanics based on two alignment conditions: the native position (Case A) and a 10 mm lateral tibial tuberosity transfer (Case B). Each of the seven analysis conditions represented three trochlear morphologies in each alignment condition (by varying lateral trochlear inclination (LTI) angle (1 to 3)). Simulations were executed in ANSYS Mechanical v24 R1 (ANSYS Inc., Canonsburg, PA, USA). The study was parametric: we could change one aspect of each case (tibial tuberosity position or trochlear inclination) while other cases remained constant.

### 2.2. Model Generation

#### 2.2.1. Image Acquisition and Segmentation

We created the subject-specific anatomical model from high-resolution helical CT data of a healthy male lower limb (slice thickness 0.625 mm, in-plane resolution 0.39 × 0.39 mm) with the approval of the institution’s ethics committee (No. 56457/7 November 2022). We segmented the cortical bone, cancellous bone, and patellar cartilage in Mimics v25 (Materialise NV, Leuven, Belgium) using semi-automated thresholding, followed by region-growing, along with potentially semi-automated modifications after segmentation. The three-dimensional geometry of the femoral condyles and tibia were created without alteration from the CT data; we provided true anatomical representation, providing sample points along the entire surface.

#### 2.2.2. Surface Reconstruction and Virtual Dysplasia

Both segmentation surfaces were smoothed (Laplacian, 30 iterations) and repaired in Geomagic Wrap (3D Systems Inc., Rock Hill, SC, USA). We generated three angles of lateral inclination (LTI) of various severity based on imposing three different angles of 15°, 10°, and 5° (proximal-to-distal and posterior-to-anterior). The angles of inclination behave akin to, but are not quantitatively equivalent to, Dejour’s classification of trochlear dysplasia; therefore, each should be considered as we could parametrise the variables at a controlled level, not quantified clinical marks. For Case B we translocated the tibial tuberosity 10 mm laterally (in the coronal plane). Translocating the tibial tuberosity in the coronal plane did change the vector of the extensor, while it did not change the morphology of the trochlea.

### 2.3. Mesh Generation and Convergence

The closed-surface models were meshed in ANSYS using 10-node quadratic tetrahedral elements (SOLID187) and mean edge length for each element version was equal to 2 mm. Contact interface was modelled in ANSYS using CONTA174 (slave) and TARGE170 (master) elements. The mesh for simulations contained 9917 nodes and 2755 elements. Mesh refinement tests using edge lengths of between 1.2 and 3.0 mm were conducted, indicating <5% variation in peak contact pressure; therefore, we established convergence with an edge length of 2 mm.

### 2.4. Material Properties

All materials were modelled as homogeneous, isotropic, and linearly elastic within the physiological loading range (Table 1). Cortical and cancellous bone properties were scaled to Hounsfield units. Patellar cartilage was assigned E = 12 MPa, ν = 0.47, consistent with published values. The MPFL and patellar tendon were represented by tension-only link elements (E = 475 MPa) with a 2% pre-strain to mimic baseline ligament tension.

### 2.5. Boundary and Loading Conditions

- Flexion simulation. Knee flexion was reproduced at 30°, 60°, and 90° by imposing anterior tibial displacements derived geometrically from tibial length (ℓ = 260 mm) and flexion angle (α). These displacements represent solver-imposed boundary conditions to generate relative patella–femur positions during flexion; they should not be interpreted as literal anatomical translations of the extensor mechanism Table 2.
- Constraints. The distal tibia was fully constrained in six degrees of freedom. The femur was left unconstrained to allow physiologic patella–trochlea interaction.
- Contacts. Patellofemoral cartilage–cartilage interaction was modelled with a friction coefficient μ = 0.02 (penalty method). Ligament–bone attachments were rigidly bonded.

Solver settings. A static, non-linear solution was applied with full Newton–Raphson iteration, large-deformation OFF, and a convergence tolerance of 0.5%.

### 2.6. Outcome Variables

Primary outcome measures included (i) peak and mean patellofemoral contact pressure, (ii) contact area, (iii) peak von Mises stress in the medial patellofemoral ligament (MPFL), and (iv) lateral patellar translation and tilt. Mechanical instability was defined a priori as >5 mm lateral translation or >50% reduction in contact area. The 5 mm threshold corresponds to values widely reported in biomechanical and clinical studies as indicative of pathologic maltracking or predisposition to dislocation. The 50% contact area cut-off reflects the biomechanical principle that loss of at least half the congruent articular footprint signifies disengagement of the patella from the trochlear groove and has been employed in previous finite element and cadaveric analyses. These thresholds were adopted for consistency and comparability, but are recognised as simplifications rather than absolute clinical cut-offs.

### 2.7. Model Validation

Model credibility was tested by comparing native configuration results at 60° flexion with published cadaveric benchmarks. The simulated peak pressure (3.6 MPa) was within 9% of experimental data, supporting validity. Simulated MPFL strain patterns qualitatively matched MRI-based patellar tracking studies, further corroborating physiological relevance.

### 2.8. Sensitivity Analysis

A one-at-a-time sensitivity analysis was performed to test robustness of the model. Cartilage modulus, ligament modulus, and friction coefficient were each perturbed by ±15% from baseline values. Peak patellofemoral pressure changed by less than 6% across all scenarios, indicating stability of the numerical framework to material property uncertainty. Although the model was based on a single anatomical specimen, these results suggest that moderate inter-subject variability in cartilage thickness, ligament stiffness, or frictional behaviour is unlikely to alter the main qualitative findings. Nevertheless, validation in multi-subject geometries remains an important priority for future work.

### 2.9. Statistical Treatment

For each alignment–dysplasia–flexion combination, three independent solves were executed with random mesh seed variations; mean ± SD values were analysed. Differences between cases were assessed with one-way ANOVA followed by Bonferroni correction (α = 0.05) in SPSS v29.

## 3. Results

### 3.1. Mesh Behaviour and Solver Convergence

The final discretisation, consisting of 9917 nodes and 2755 ten-node SOLID187 elements, proved numerically stable. Reducing the characteristic edge length from 3 mm to 2 mm changed the peak patellofemoral contact pressure by less than five percent, thereby satisfying the pre-defined convergence criterion. Throughout the Newton–Raphson iteration no element distortion, over-constraint, or abnormal residuals were encountered, confirming solution robustness.

### 3.2. Global Kinematics

**Figure 1:**
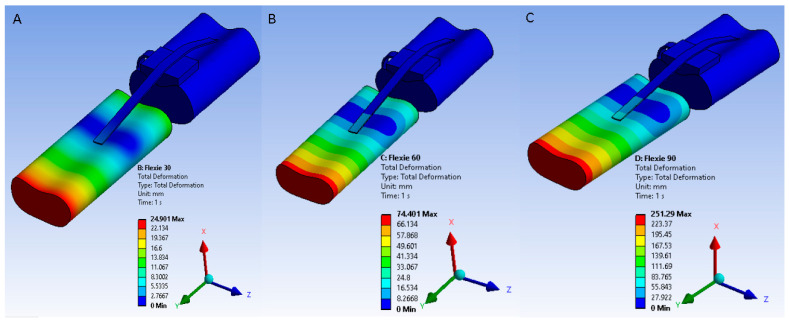
Solver-imposed tibial displacements used to reproduce knee flexion at 30°, 60°, and 90°. Displacement fields are illustrated for (**A**) 30°, (**B**) 60°, and (**C**) 90° flexion. These values represent boundary-condition translations applied within the solver, not literal anatomical excursions. Under these imposed conditions, the patella migrates distally along the trochlear groove in a manner consistent with physiological tracking.

Solver-imposed tibial displacements required to reproduce knee flexion were 23.6 mm at 30°, 70.9 mm at 60°, and 240 mm at 90°. These values represent boundary-condition translations applied to the tibia, not literal anatomical excursions of the extensor mechanism. Under these imposed motions, the patella migrated distally along the femoral trochlea in a manner consistent with physiological patellar tracking. Figure 1 illustrates the resulting displacement fields across the extensor mechanism.

### 3.3. Von Mises Stress Distribution

**Figure 2:**
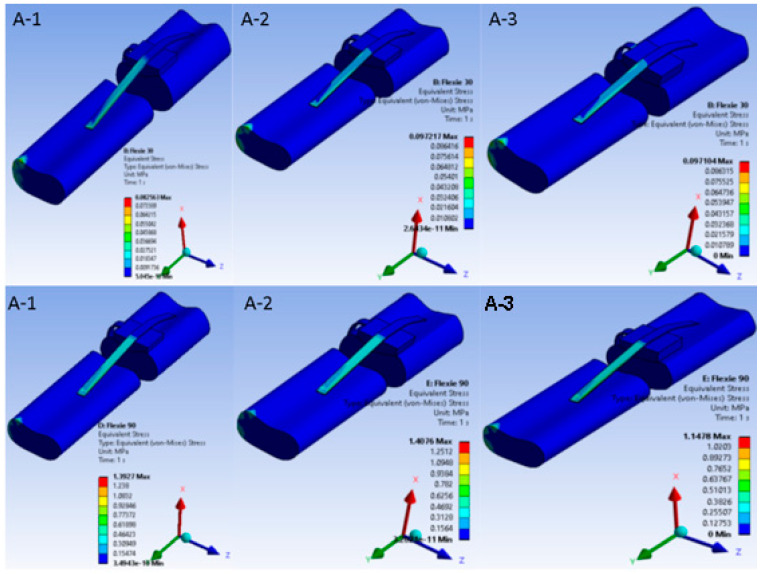
Equivalent von Mises stress in the extensor mechanism under native alignment. Von Mises stress distribution in the native-alignment model. Heat maps demonstrate stress patterns in patellar cartilage and the medial patellofemoral ligament (MPFL) across three trochlear inclination angles: (**A-1**) = 15°, (**A-2**) = 10°, (**A-3**) = 5°. Rows correspond to flexion at 30° and 90°. As inclination decreases, cartilage stresses intensify and progressively transfer to the MPFL, with maximal concentration on the medial patellar facet and proximal MPFL fibres at 90° in (**A-3**)—replicating the load shift from osseous to soft-tissue stabilisers seen in severe trochlear dysplasia.

Under native alignment (Case A) a progressive decrease in trochlear lateral inclination from 15° (A-1 to 5° (A-3) produced steadily higher von Mises stresses in both patellar cartilage and the MPFL at every flexion angle. Maximal values occurred in configuration A3 at 90° flexion, where stress concentrated on the medial patellar facet and proximal fibres of the MPFL (Figure 2). This pattern reflects progressive transfer of load from cartilage to ligamentous structures as trochlear containment weakens, consistent with the clinical presentation of instability in severe dysplasia.

**Figure 3:**
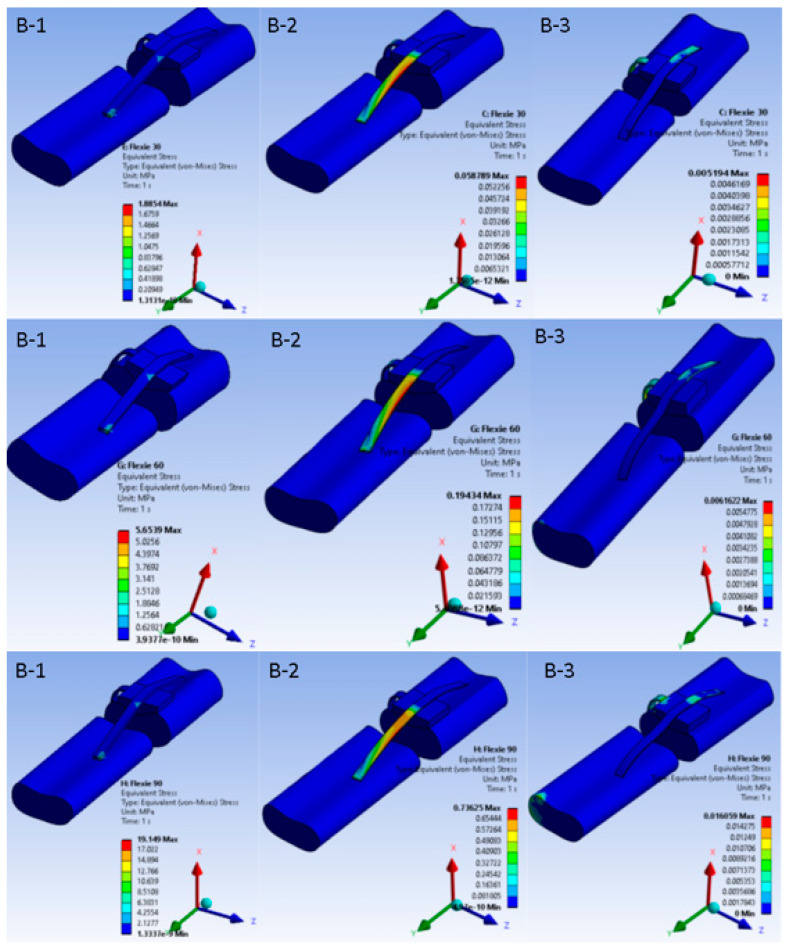
Equivalent von Mises stress in the extensor mechanism after 10 mm lateral tibial tuberosity transfer. Nine heat-map panels display the von Mises stress field for the lateralised-tuberosity configuration (Case B). Columns correspond to decreasing lateral-trochlear inclination—(**B-1**) = 15°, (**B-2**) = 10°, (**B-3**) = 5°—and rows to progressive knee flexion (30°, 60°, 90°). All sub-images share a unified colour bar (0–10 MPa). At 30° flexion stresses in (**B-1**) concentrate around the patellar tendon insertion; as inclination diminishes and flexion deepens, the high-stress locus migrates medially into the MPFL and adjacent retinacular structures. In (**B-3**) at 90° the patella has partly disengaged from the groove; cartilage stresses drop and peak values reside almost exclusively within the soft tissues—illustrating the load shift associated with combined trochlear shallowing and tibial tuberosity lateralisation.

When the tibial tuberosity was shifted 10 mm laterally (Case B) the principal stress locus changed. In variant B1 (15° inclination) stresses accumulated chiefly around the patellar tendon insertion on the tibia. By contrast, in variant B3 (5° inclination) the highest stresses migrated to the medial ligamentous structures as the patella began to disengage from the trochlear groove, a pattern illustrated in Figure 3.

### 3.4. Contact Mechanics

**Figure 4:**
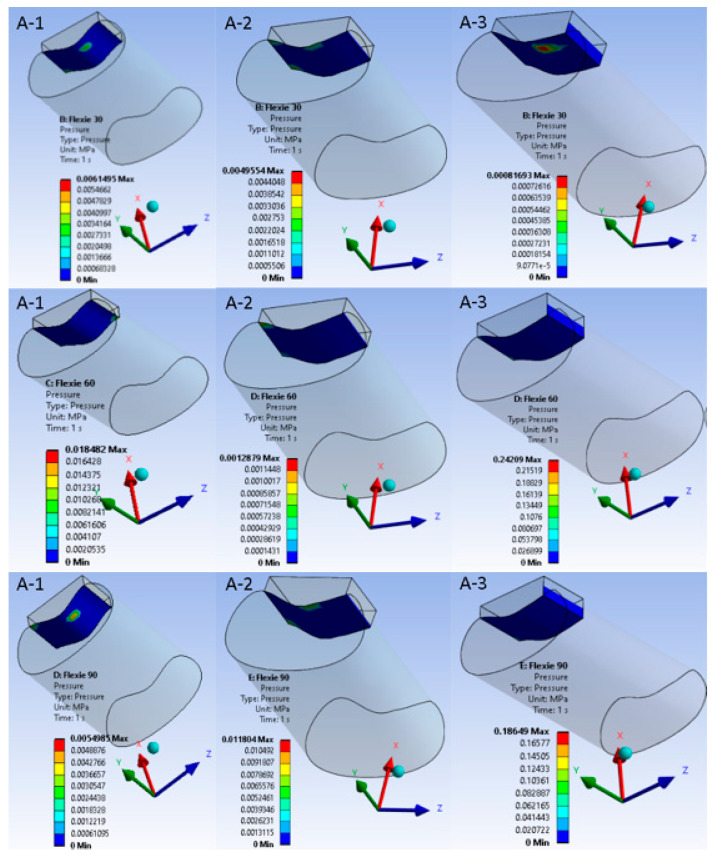
Patellofemoral contact pressure distribution under native alignment (Case A). Columns represent trochlear inclination angles ((**A-1**) = 15°, (**A-2**) = 10°, (**A-3**) = 5°); rows correspond to flexion at 30°, 60°, and 90°. As flexion deepens, the patellar contact patch migrates distally along the trochlea and widens. Shallower inclinations disperse load over a broader area, lowering mean pressure but increasing the demand on soft-tissue stabilisers.

**Figure 5:**
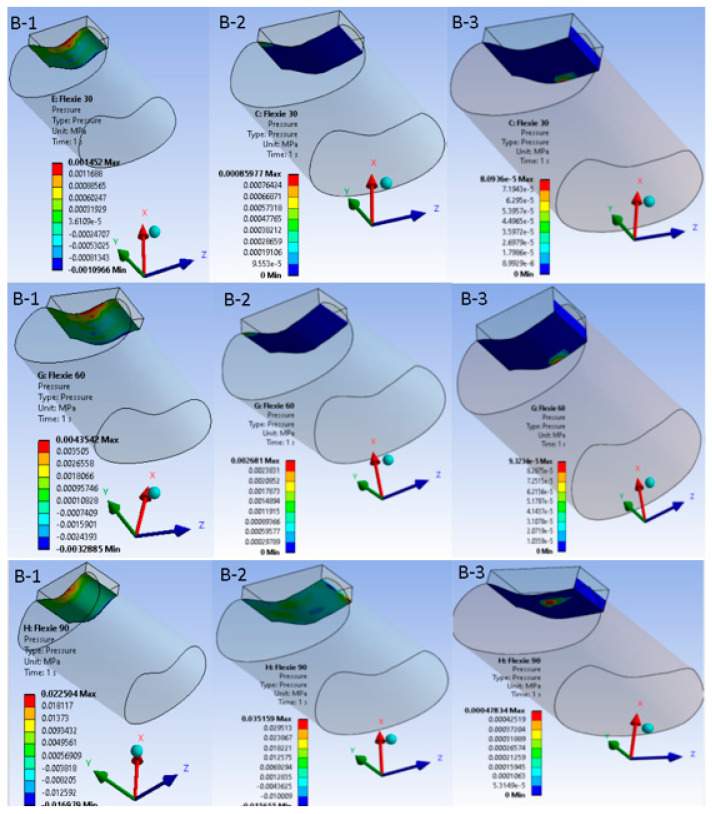
Patellofemoral contact pressure distribution after 10-mm lateral tibial-tuberosity transfer. Equivalent pressure maps for Case B are displayed with the same colour scale and layout as Figure 4. At 30° flexion (**B-1**), lateralisation produces an eccentric, crescent-shaped footprint that is already shifted laterally compared to the native configuration. At 60° flexion (**B-2**), this lateral displacement persists with lower peak pressures, indicating partial offloading of the joint surface. At 90° flexion (**B-3**), however, only a narrow rim of cartilage remains engaged, underscoring the trade-off between reduced peak load and loss of congruent contact.

Contact behaviour varied systematically with both flexion angle and trochlear geometry. At 30° of flexion the contact patch was smallest in variants A1 and B1; as flexion increased and/or lateral inclination diminished, the area broadened, reaching its widest extent at 90° in A3. Because the contact patch broadened with increasing flexion and shallower inclination, mean contact pressure fell from A1 through A3. The same trend appeared in the B-series, although absolute pressures were consistently lower due to the eccentric footprint created by lateral tibial tuberosity placement. In the most extreme case (B3, 5° inclination at 90° flexion), congruent patellofemoral contact was largely lost, leaving only a narrow rim of cartilage engaged, effectively simulating the biomechanical analogue of a lateral dislocation. Figure 4 and Figure 5 portray these pressure maps and highlight the inverse relation between contact area and pressure magnitude.

### 3.5. Frictional Shear

**Figure 6:**
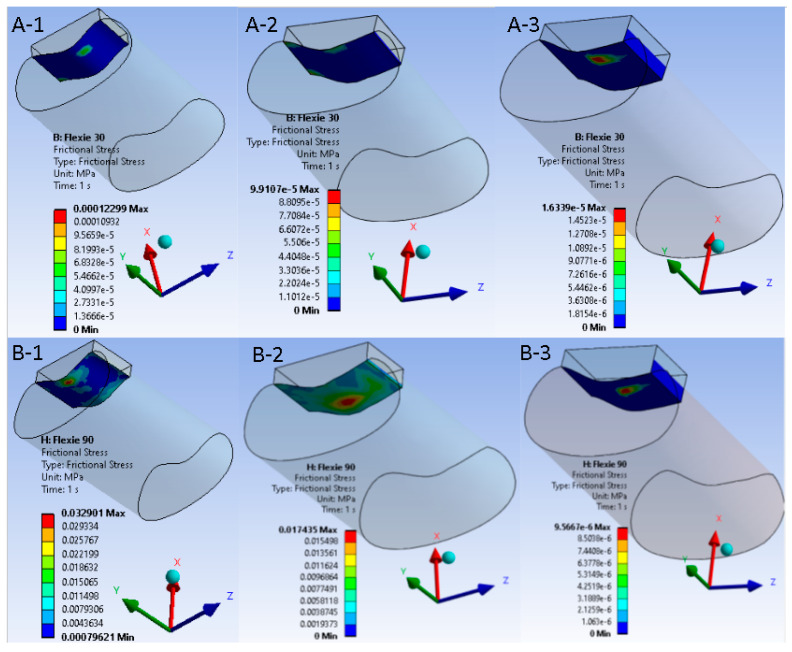
Frictional shear maps at early (30°) and deep (90°) flexion for native versus lateralised alignment. Upper panels ((**A-1**)–(**A-3**)) display frictional-stress magnitude in the native tibial tuberosity configuration (Case A) at 30° of knee flexion; lower panels ((**B-1**)–(**B-3**)) show the same variable after 10 mm tibial tuberosity lateralisation (Case B) at 90° of flexion. Within each row the trochlear lateral inclination decreases from 15° to 5°, corresponding to model variants “1”, “2”, and “3”. A unified colour bar (units: MPa) is applied to all six sub-figures. In the native alignment, frictional shear is modest and declines progressively as the contact patch expands ((**A-1**)–(**A-3**)). After lateralisation, shear is initially higher but falls precipitously with decreasing inclination; at 5° ((**B-3**)) shear approaches zero, reflecting appreciable de-planing of the cartilage surfaces and transfer of load to the soft tissues, the mechanical analogue of a lateral patellar dislocation.

Frictional shear followed the pressure field. Within Case A it declined gradually from A1 to A3 as the enlarged contact patch dissipated the tangential load. In the lateralised tibial tuberosity models, the shear component dropped more abruptly; variants B2 and B3 approached zero once appreciable de-planing of the cartilage surfaces occurred. This behaviour is summarised in Figure 6, which juxtaposes a representative 30° panel with a 90° panel for each alignment condition.

### 3.6. Key Numerical Observations

Quantitatively, the native model at 60° flexion produced a peak contact pressure of 3.6 MPa, which lay within 9% of published cadaveric benchmarks, supporting model validity. Lateralisation of the tibial tuberosity reduced mean pressure by ~25%, but when combined with shallow trochlear slopes (≤8°) it permitted lateral patellar translations exceeding 5 mm, a threshold generally regarded as mechanically unstable. Frictional shear at the interface dropped below 0.02 MPa once the contact area fell below half of the native footprint, numerically reproducing the loss of congruent engagement that characterises clinical dislocation.

## 4. Discussion

The finite element analyses presented here illuminate the coupled influence of trochlear morphology and tibial tuberosity (TT) position on patellofemoral mechanics. When the lateral trochlear inclination (LTI) was reduced from 15° to 5° under native alignment, the patella drifted laterally, the contact patch enlarged, and the mean articular pressure fell, yet the peak von Mises stress in the medial patellofemoral ligament (MPFL) rose markedly. Because the MPFL is the principal restraint in the first 20–30° of knee flexion, its higher tensile load explains why patients with severe dysplasia often complain of giving-way rather than pain. Lateralising the TT by 10 mm amplified this effect; at an LTI of 5° the patella disengaged from the trochlea by 60° of flexion, frictional shear at the cartilage interface collapsed, and load was shunted to the soft tissues, numerically reproducing a clinical lateral dislocation.

Model credibility is supported by the close agreement between our native 60°flexion peak pressure (3.6 MPa) and the 3.0–4.0 MPa range measured in vivo with weight-bearing MRI by Besier and colleagues [7]. The alignment-dependent redistribution of cartilage load that we observed is also consistent with cadaveric studies in which experimentally increased Q-angles widened the contact area and displaced it laterally, although those investigations retained a normal trochlear depth and therefore detected rising, not falling, pressures once maltracking became extreme.

Comparative computational work provides further context. Kleeman-Forsthuber et al. combined a clinical case–control series with finite element simulation and reported that an anatomic patellar component generated markedly higher strain energy density at the superior pole of the patella than a medialised dome design, a finding that paralleled their clinical observation of anterior knee pain, effusions, and fragmentation in the anatomic group [8]. Our results complement and extend those data: where Kleeman-Forsthuber et al. identified a component-specific shift in bony stress, we demonstrate that, once the lateral trochlear inclination falls below about eight degrees, the destabilising moment produced by a lateralised tibial tuberosity overrides even the load-spreading advantage of a large contact area, allowing the patella to disengage despite apparently modest intra-articular pressures. Elias et al. [9], using dynamic simulation, found that trochleoplasty increased peak contact pressures whereas untensioned MPFL grafts could unload cartilage at the expense of stability [9]; the same pressure–stability trade-off is evident in our native-alignment models, in which restoring a steeper LTI preserved containment but concentrated stress on a smaller footprint.

The thresholds applied in the present work are supported by both biomechanical and computational evidence. A recent study emphasised that the MPFL provides the majority of restraint against lateral patellar translation, but its capacity is exceeded once displacement reaches only a few millimetres, predisposing the patella to recurrent instability [9,10]. Computational modelling by Elias et al. similarly showed that lateral translations beyond approximately 5 mm exceed the restraint capacity of the MPFL and generate maltracking unless corrected by bony realignment [9]. For articular congruence, Kaiser et al. demonstrated using finite element analysis that trochleodysplastic knees exhibit significantly reduced patellofemoral contact areas compared with healthy controls, and that sulcus-deepening surgery further reduces contact footprint. These findings support the use of a >50% reduction in contact area as a biomechanical surrogate for patellar disengagement from the trochlear groove. Taken together, this literature provides a rationale for adopting >5 mm translation and >50% contact area loss as operational definitions of mechanical instability in the current study, while recognising that these remain simplified criteria requiring validation across larger cohorts [4].

Recent systematic evidence strengthens the argument for quantitative, patient-tailored planning in patellofemoral surgery. Contemporary algorithms for lateral patellar instability were studied and concluded that outcomes are optimised only when the chosen procedure matches the full profile of osseous and soft-tissue risk factors, yet they noted that surgeons still lack objective tools to balance competing mechanical goals at the point of care [11]. Tan et al. [12], in a meta-analysis of 546 patella alta knees, showed that distal-based tibial tubercle procedures correct patellar height and TT–TG distance more effectively than isolated proximal reconstructions, but these benefits are offset by a higher incidence of secondary surgery; the authors therefore called for decision-support methods capable of quantifying the trade-off between alignment gains and procedural morbidity [12]. Complementing these data, Pavone et al. compared surgical and conservative strategies after first-time dislocation and found a five-fold reduction in dislocation and significantly better Kujala scores with operative treatment, while emphasising the persisting need for high-level evidence to guide individual case selection [13]. The finite element framework presented in the current study responds directly to these gaps, allowing surgeons to visualise how specific combinations of trochlear morphology and tibial tuberosity position influence both contact pressure and mechanical stability, and thereby to tailor combined bony and soft-tissue interventions more precisely to each dysplastic knee. Another clinical study further supports the need for personalised treatment strategies showing that inadequate between the morphology of the pathology and fixation strategies can lead to suboptimal outcomes [14].

The clinical implications are two-fold. Firstly, reliance on the TT–TG distance alone is inadequate. Classical algorithms recommend mTTO when TT–TG exceeds 20 mm, yet our data indicate that in knees with LTI ≤ 8° even “borderline” values around 15 mm permit large lateral translations without a compensatory rise in cartilage pressure to alert the surgeon or patient. Secondly, recent anatomical–biomechanical work underscores that a graft placed in isolation cannot compensate for deficient bony containment. Tanaka et al. [15] showed that the medial patellofemoral ligament (MPFL) supplies, on average, only about one-half of the resistance to lateral patellar translation, the remainder being furnished by the medial quadriceps tendon–femoral ligament, the medial patellotibial ligament, and the medial patellomeniscal ligament, collectively termed the medial patellofemoral complex (MPFC). When the osseous chassis is inadequate, or when the graft is fixed outside the broad “cloud” of native femoral attachment points, tension concentrates within the reconstructed MPFL, pre-tensioning it throughout the flexion arc and predisposing the tissue to elongation, loss of isometry and eventual recurrence of instability. The >40% rise in MPFL stress recorded in our unstable models mirrors this mechanism and reinforces the recommendation that successful soft-tissue reconstruction must be paired with restoration of anatomic containment and, where necessary, augmentation of the distal medial restraints of the MPFC [10,15,16]. Combining MPFL reconstruction with bony realignment therefore appears prudent whenever trochlear depth or TT position is abnormal.

There are following limitations of the current finite element study. First, even though the meanderings of the femoral condyles, patella, and tibia were reproducibly obtained from high-quality CT data, smoothing techniques and consequent Boolean operations used to create the trochlear variants did lead to some simplification of the original structures. The resulting models likely did not completely resemble the complex three-dimensional surface curvatures affecting patellofemoral tracking. Second, a measure of knee flexion was simulated with quasi-static anterior tibial displacements, informed by tibial length and flexion angle. Otherwise, as we developed them for consistent simulation of comparative femur–patella–tibia positions at 30°, 60°, and 90°, restoring reasonable three-dimensional movement was not possible. Thus, the models would fail to take into account the effect of forces from all quadriceps muscle heads and/or any secondary stabilisers affecting patellar tracking as they appear to be mobilised during useful activities. Likewise, tibial displacement imposed by the solver on the tibial reference position should be treated only as boundary conditions by the model and should not be interpreted as equivalent translational displacements of the extensor mechanism. Third, morphology of the trochlea was parameterised through changes in the lateral inclination angle to values of 15°, 10°, and 5°. Although these angles mirror the gradual shaping associated with Dejour grades A–C, they do not correspond to the entirety of radiographic landmarks used in the formal Dejour classification. Therefore, it is important to assess our results as being induced angle-based parametric variations, rather than simulations of clinical grades of Dejour’s classification. Fourth, the tibial tuberosity lateralisation was modelled without a direct calculation of the tibial tuberosity–trochlear groove (TT–TG) distance. Although TT position and TT–TG distance have a close association with respective anatomical relationships, hence precautions for extrapolating our parameters to established clinical TT–TG cut-offs should be exercised. Fifth, the material properties were idealised homogeneous, isotropic and linearly elastic, and was approximated for uniform cartilage thickness. It was not constructed to express time-dependent viscoelasticity or inter-subject variability. Similarly, ligament modelling was simplified to tension-only structural elements conditioned to the anatomical footprints; while this did reproduce gross deformation patterns, it did not permit a more precise modelling of fibre-bundle recruitment or interactivity of the MPFL position throughout flexion. Although the study was completed on a single individual, provided that a sensitivity analysis indicated that moderate variations in cartilage stiffness, ligament stiffness, and friction behaviour did not substantially affect the results, confidence can be inferred for the trends observed, and this does not replace the multiple anatomical validation. The figures were developed from engineering software output that emphasise stress distribution depiction, although the legends and annotations were addressed to potentially infer clinical relevance; this may differ value from conventional radiological or intraoperative images. Regardless of acknowledged limitations, the model was validated against cadaveric benchmarks, and the parametric composition of the design provided an unambiguous interpretation for understanding how the slope of the trochlea and position of the tibial tuberosity interact to affect contact pressure and patellar instability.

## 5. Conclusions

This finite element investigation delineates how trochlear morphology and tibial tuberosity position jointly govern patellofemoral mechanics. A native trochlea with a steep lateral wall maintained congruent tracking and moderate pressures, whereas progressive loss of lateral inclination enlarged the contact patch, dispersed articular load, and transferred the restraint burden to the medial patellofemoral ligament. Lateralisation of the tibial tuberosity accentuated this imbalance: mean pressure fell by roughly one quarter, yet once the trochlear slope dropped below about eight degrees the patella disengaged from the groove, frictional shear collapsed, and peak stress migrated into the soft tissues, reproducing the biomechanical signature of clinical dislocation. The model’s native-pressure values agree closely with published cadaveric data, supporting its validity, and the systematic parametric design exposes quantitative thresholds that can inform surgical planning.

Taken together, the results argue that the traditional tibial tuberosity–trochlear groove distance should not be interpreted in isolation. Where trochlear lateral inclination is shallow, medialisation osteotomy may be warranted even at “borderline” distances, and groove deepening should be considered despite the modest rise in cartilage pressure that it entails. Conversely, a sufficiently steep trochlea can tolerate small increases in extensor vector offset without jeopardising stability, allowing less invasive, cartilage-sparing procedures to suffice. By integrating these geometric variables into a single, validated computational framework, the present study provides clinicians with a clearer biomechanical rationale for tailoring combined bony and soft-tissue interventions to the individual dysplastic knee.

## Figures and Tables

**Table 1:** Material proprieties of the model used.

Component	Elastic Modulus (E)	Poisson’s Ratio (ν)	Notes
Cortical bone	17,000 MPa	0.30	Scaled to Hounsfield units (typical)
Cancellous bone	500 MPa	0.30	Typical trabecular bone properties
Patellar cartilage	12 MPa	0.47	Based on literature assumptions
MPFL and Patellar tendon	475 MPa	-	Tension-only, pre-strain of 2%

**Table 2:** Flexion angles for the model used.

Flexion Angle	Tibial Anterior Translation (mm)
30	23.6
60	70.9
90	240

## Data Availability

The data presented in this study are available on request from the corresponding author due to privacy and legal reasons.
